# Bacterial co-infections and secondary infections and their antimicrobial resistance in Covid-19 patients during the second pandemic wave

**DOI:** 10.3205/dgkh000465

**Published:** 2024-03-05

**Authors:** Ruchita Attal, Vijayshri Deotale

**Affiliations:** 1 Dept of Microbiology, Mahatma Gandhi Institute of Medical Sciences, Sevagram, Wardha, Maharashtra, India

**Keywords:** COVID-19, bacterial co-infection, spectrum of pathogens, antimicrobial resistance

## Abstract

**Background:**

COVID-19 pneumonia with an unusual outbreak is considered a new, global public health threat. Microbiological characterization of co-infections in patients with COVID-19 is important, and antimicrobial use is high. We aimed to describe microbiologically confirmed co-infections and the antimicrobial resistance of the causative pathogens.

**Method:**

From January to December 2020, we tested 1,301 patients who were COVID-19 positive. We received clinical samples (blood, respiratory and sterile body fluids) of COVID-19 patients who were suspected to have bacterial co-infections. Samples were processed and antimicrobial susceptibility testing was performed based on the CLSI recommendation. Demographic, clinical, laboratory and outcome data of those with positive cultures were collected.

**Result:**

A total of 1301 COVID-19 patients (568 from the COVID ward and 733 from ICU) were admitted to the Covid care ward of a tertiary care hospital. 363 samples were sent for culturing and testing antibiotic susceptibility, of which 131 (36%) were found to be culture-positive (90 from ICUs, 41 from wards). Out of the 143 total isolates thus obtained from 131 samples, the majority (62.2%) were Gram-negative bacteria, and most of them were (70.8%) multidrug resistant.

**Discussion:**

Bacterial co-infection in patients with COVID-19 is more commonly reported in the severely ill hospitalized individuals (58%), particularly in the ICU (73.3%) setting. In terms of mortality, almost half of co-infected patients died (51.1%). In most of them, the cause of death was found to be sepsis with post-COVID ARDS (58%).

**Conclusion:**

Co-infection in COVID-19 patients may affect the outcome in terms of increasing the hospital stay.

## Zusammenfassung

**Hintergrund:**

Die COVID-19-Pneumonie ist eine neue, weltweite Bedrohung für die öffentliche Gesundheit. Die mikrobiologische Charakterisierung von Koinfektionen bei Patienten mit COVID-19 ist wichtig und der Einsatz von Antibiotika ist hoch. Unser Ziel war es, mikrobiologisch bestätigte Koinfektionen und die Antibiotikaresistenz der verursachenden Erreger zu beschreiben.

**Methode:**

Von Januar bis Dezember 2020 wurden bei 1.301 an COVID-19 erkrankten Patienten mit Verdacht auf eine bakterielle Koinfektion klinische Proben (Blut, respiratorische und sterile Körperflüssigkeiten). Die Proben wurden aufbereitet und die Empfindlichkeit gegenüber Antibiotika auf der Grundlage der CLSI-Empfehlung getestet. Für die Patienten mit positiver bakterieller Kultur wurden demografische, klinische, Labor- und Verlaufsdaten erfasst.

**Ergebnisse:**

Insgesamt wurden 1.301 COVID-19 Patienten (568 auf die COVID-Station, 733 auf die Intensivstation) in das Krankenhaus der Tertiärversorgung aufgenommen. Von 363 untersuchten Proben waren 131 (36 %) kulturell positiv (90 von der Intensivstation, 41 von der COVID-Station). Von 143 Isolaten aus 131 Proben waren 62,2% Gram-negative Bakterien; davon waren 70,8% multiresistent.

**Diskussion:**

Bakterielle Koinfektionen bei Patienten mit COVID-19 wurden häufiger bei schwer kranken Krankenhauspatienten (58%) insbesondere auf der Intensivstation (73,3%) festgestellt. 51,1% der Patienten mit einer Koinfektion verstarb. Bei 58% war die Todesursache eine Sepsis mit post-COVID ARDS (58%).

**Schlussfolgerung:**

Eine Koinfektion kann bei Patienten mit COVID-19 den Krankheitsverlauf verschlechtern und den Krankenhausaufenthalt verlängern.

## Introduction

The SARS–CoV-2 disease has become a public health challenge. SARS-CoV-2 pneumonia was termed “COVID-19” by the WHO on 11^th^ Feb 2020 [1], declaring the novel coronavirus outbreak a public health emergency of international concern [2]. The co-infection/secondary bacterial infections (SBI) of SARS-CoV-2 with microorganisms raise further difficulties of diagnosis, treatment, and prognosis. Co-infection in patients with severe influenza has been reported to be as high as 20–30% [3], [4]. This is associated with a greater severity of illness, greater use of healthcare resources, and increased risk of death. The prevalence, incidence and characteristics of bacterial infection in patients infected with SARS-CoV-2 is not well understood and has been identified as an important knowledge gap [5], [6]. However, little is known about the mechanism by which a virus can predispose the patient to develop a secondary infection which results in longer ICU stays and in-hospital mortality. Further, before the microbiological confirmation of SBIs, the clear majority of COVID-19 patients were given empirical antimicrobial treatment [7]. Hence, it is expected that this increased overuse of empirical antibiotics without their actual need will lead to the emergence of antimicrobial resistance in the future, limiting treatment options to higher class of antibiotics such as colistin and tigecycline.

### Aims and objective

Although the burden of Covid co-infection is higher in our developing country, such active surveillance is not being conducted in any large tertiary-care rural hospital in central India. With this background, this study was conducted to determine the etiology and antimicrobial resistance profile of co-infections (at the time of admission) or secondary bacterial infection 48 hrs after hospital admission and to document the subsequent clinical outcomes in hospitalized COVID-19 patients, in order to use this evidence to guide optimal antimicrobial use in COVID-19 patients.

## Method

### Type of study

Observational cross-sectional study.

### Setting

COVID-19 ward and ICU of rural hospital in central India.

### Inclusion criteria

All COVID-19 patients with a suspected co-infection or SBI during the study period with positive RT-PCR and clinically positive results from samples collected within 2 days of admission were categorized as co-infection, and those collected more than 2 days after admission as secondary infections.

### Exclusion criteria

COVID-19 RT-PCR negative patients, COVID-19 RT-PCR positive Outdoor Patients Department (OPD) patients and culture results recorded as mixed growth or contaminated were excluded.

### Implementation

The study was performed during April–July 2021 (2^nd^ wave of the pandemic). Clinical samples consisted of blood, respiratory and sterile body fluids of COVID-19 patients were collected according to recommendations in the personal protective equipment guidelines. The total number of analyzed samples studied was 363. Samples were processed and evaluated by conventional methods. The growth of bacterial colonies was confirmed by Gram staining and standard biochemical testing. Antimicrobial susceptibility testing was performed based on the CLSI recommendation. As per clinician request the VITEK ID/ AST test system was implemented [8]. Demographic, clinical, laboratory and outcome data of those whose culture was positive were collected, e.g., date of admission, date of culture-positive results, antimicrobial susceptibility profile of isolates, length of hospital stay and patient outcome. 

## Results

### Demographic Details

At our COVID-care facility, 1,301 COVID-19 patients were admitted during the second pandemic wave, out of which 733 (56%) were admitted to the ICU and 568 (44%) to the COVID ward. 

During this period, we received 363 clinical specimens (28%): 163 blood cultures, 156 urine cultures, 30 endotracheal aspirates, 9 cerebrospinal fluid samples (CSF) and 5 of pleural fluid, collected and processed using the recommended personal protective equipment guidelines (Table 1 (Tab. 1)). Repeat samples were excluded if similar results were obtained. Also, culture results showing mixed growth or contaminants were excluded. Among these 363 clinical specimens 210 were from the COVID ICU and 153 from the COVID ward.

Overall, 131 cultures were positive (36.1%) and were included, documenting the corresponding demographic data, severity of illness based on ICU admission, outcome measures and length of stay (Table 2 (Tab. 2)). Out of 131 cultures positive samples, 55.7% had a positive blood culture, 13.7% had a positive respiratory culture, 29% had a postivie urine culture, and sterile body fluid cultures were positive in 1.5% patients. 73 (44.7%) of the 163 blood-culture specimens, 38 (24.3%) of the 156 urine specimens, 18 (60%) of the 30 respiratory and 2 (14.3%) of 14 sterile body fluid specimens were positive for bacterial infection (Table 1 (Tab. 1)).

### Severity of illness and outcome measures

This was based on admission to ICU and hospital mortality. Out of 1301 total COVID-19 admissions during the second wave, 733 required (56%) ICU admission; of these, 90 (68.7%) were positive for bacterial infections. Overall mortality was 70 (5.4%) among 1,301 patients on the COVID-19 war and 7.6% among the 733 ICU patients.

### Etiological profile of secondary infections/co-infections

A total of 363 clinical specimens (blood, respiratory, urine and sterile body fluids) were received for microbiological culture, of which 232 (64%) were culture-negative. Overall, 131 (36%) were culture-positive (Table 3 (Tab. 3) and Table 4 (Tab. 4)). Out of these 131 patients, 73 (55.7%) had blood stream infections, 38 (29%) had urinary tract infections, and 18 (13.7%) had respiratory tract infections. 

A total of 143 significant bacterial isolates was obtained from 131 specimens. 29 coagulase-negative staphylococci and 4 *corynebacterium* species were excluded as common contaminants, due to its absence in repeated samples. The majority (62.2%) were Gram-negative organisms, i.e., 44 (49%) Enterobactericeae, 26 (29.2%) *Acinetobacter* spp., followed by *P. aeruginosa* with 19 (21.3%). Of 54 (37.8%) Gram-positive organisms, 34 (63%) were *S. aureus*. 

### Antimicrobial resistance (AMR)

**Figure 1:**
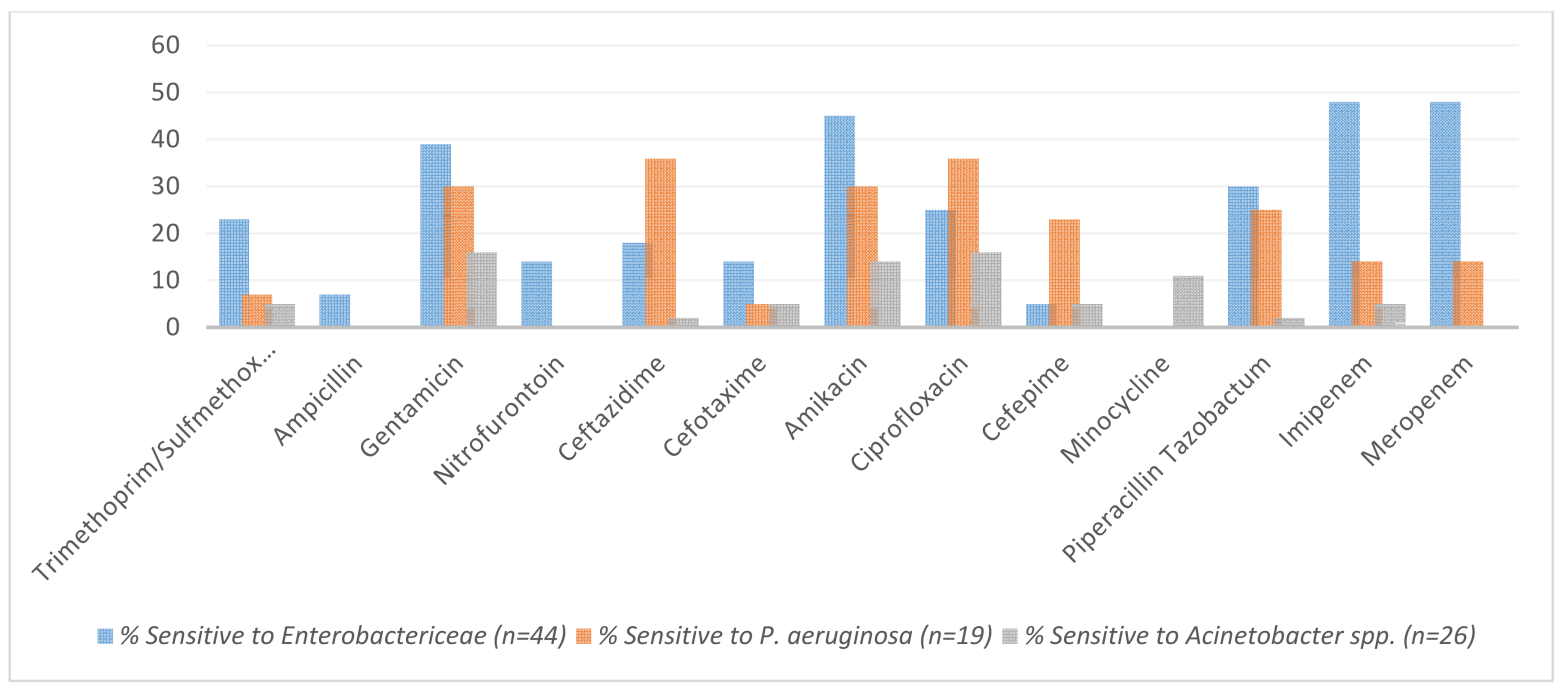
Sensitivity (%) of Gram-negative bacteria (n=89)

Figure 1 (Fig. 1) presents the percentage sensitivity of Gram-negative bacteria to antibiotics. The dataset comprises 89 cases of Gram-negative bacterial infections. The percentage sensitivity indicates the proportion of cases where the treatment effectively targeted and controlled the Gram-negative bacterial infection. This figure provides insights into the efficacy of interventions against Gram-negative organisms. 

**Figure 2:**
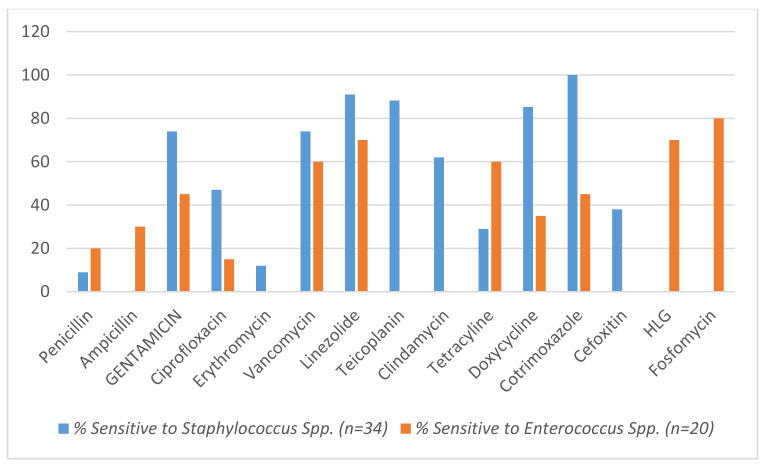
Sensitivity (%) of Gram-positive bacteria (n=54)

Figure 2 (Fig. 2) portrays the percentage sensitivity of Gram-positive bacteria in response to antibiotics. The dataset used for this analysis consists of 54 cases of infections caused by Gram-positive bacteria.

## Discussion

The co-occurrence of bacterial infections alongside COVID-19 has been a subject of significant interest and concern since the emergence of the pandemic. Bacterial co-infections can complicate the clinical course of COVID-19 patients, leading to increased morbidity and mortality. 

Table 5 (Tab. 5) presents a compilation of rates of bacterial co-infection reported in various studies conducted in different regions and time frames. The studies included in Table 5 (Tab. 5) span a wide range of time frames from the early stages of the pandemic in Wuhan to more recent investigations in 2021. They were conducted in diverse geographical locations, including India, Italy, and Wuhan (China). The studies varied in their sample sizes with patient numbers ranging from hundreds to several thousand. The reported incidence of bacterial co-infections in COVID-19 patients shows considerable variability across studies (Table 5 (Tab. 5)). The rates of bacterial co-infection ranged from as low as 6.8% to as high as 20% [8], [9]. This wide range could be attributed to various factors, such as differences in study populations, patient demographics, healthcare practices, and diagnostic criteria for identifying bacterial infections.

Geographical differences might contribute to variations in bacterial co-infection rates. For instance, studies conducted in India [8], [10] as well as our study report rates between 7.9% and 13%; in Italy [5] reported an 11% incidence (Table 5 (Tab. 5)). These differences could reflect variations in healthcare infrastructure, clinical management protocols, and prevalence of bacterial pathogens in different regions.

There appears to be no consistent temporal trend in bacterial co-infection rates over the course of the pandemic. Studies conducted early in the pandemic report lower rates compared to those conducted later [10], [11], [9] as does our study. This disparity might be influenced by changing clinical practices, evolving understanding of the disease, and variations in patient populations.

Several limitations should be acknowledged when interpreting the findings from Table 5 (Tab. 5). The differences in study designs, patient populations, and diagnostic methodologies can introduce biases. The definition of bacterial co-infection might also vary between studies, leading to inconsistencies in reported rates. Additionally, some studies did not provide details on specific bacterial pathogens involved, which hinders a comprehensive analysis of the types of infections.

Understanding the rates of bacterial co-infection in COVID-19 patients is crucial for optimizing patient management and healthcare resource allocation. The variability in reported rates underscores the need for standardized diagnostic criteria and protocols for identifying bacterial co-infections in COVID-19 cases. Further research is warranted to explore factors contributing to regional and temporal disparities and to investigate the impact of bacterial co-infections on patient outcomes.

## Conclusion

The findings, presented in Table 5 (Tab. 5), highlight the diverse landscape of bacterial co-infections in COVID-19 patients across different studies. The variations in reported rates emphasize the importance of cautious interpretation and the need for robust research methodologies to better understand the complex relationship between bacterial co-infections and COVID-19. In India, treatment guidelines for HAIs have been issued by the Indian Council of Medical Research, based on our indigenous antimicrobial resistance data. Antimicrobial stewardship programs must focus on supporting the optimal selection of empiric treatment and rapid de-escalation, based on culture reports. 

## Notes

### Author’s ORCID

- Ruchita Attal: 0000-0003-1766-4682
- Vijayshri Deotale: 0000-0002-9347-9836

## Figures and Tables

**Table 1:**
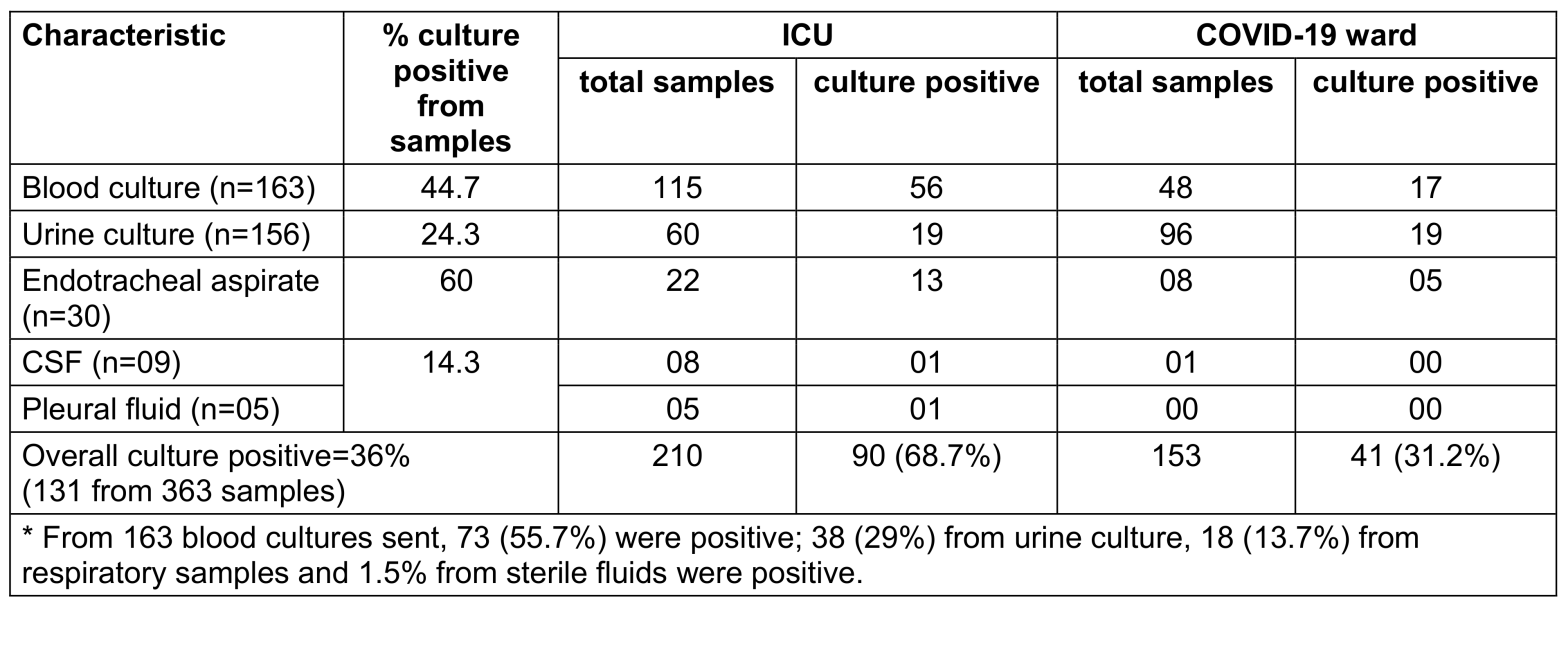
Distribution of samples according to growth of organisms (n=363)*

**Table 2:**
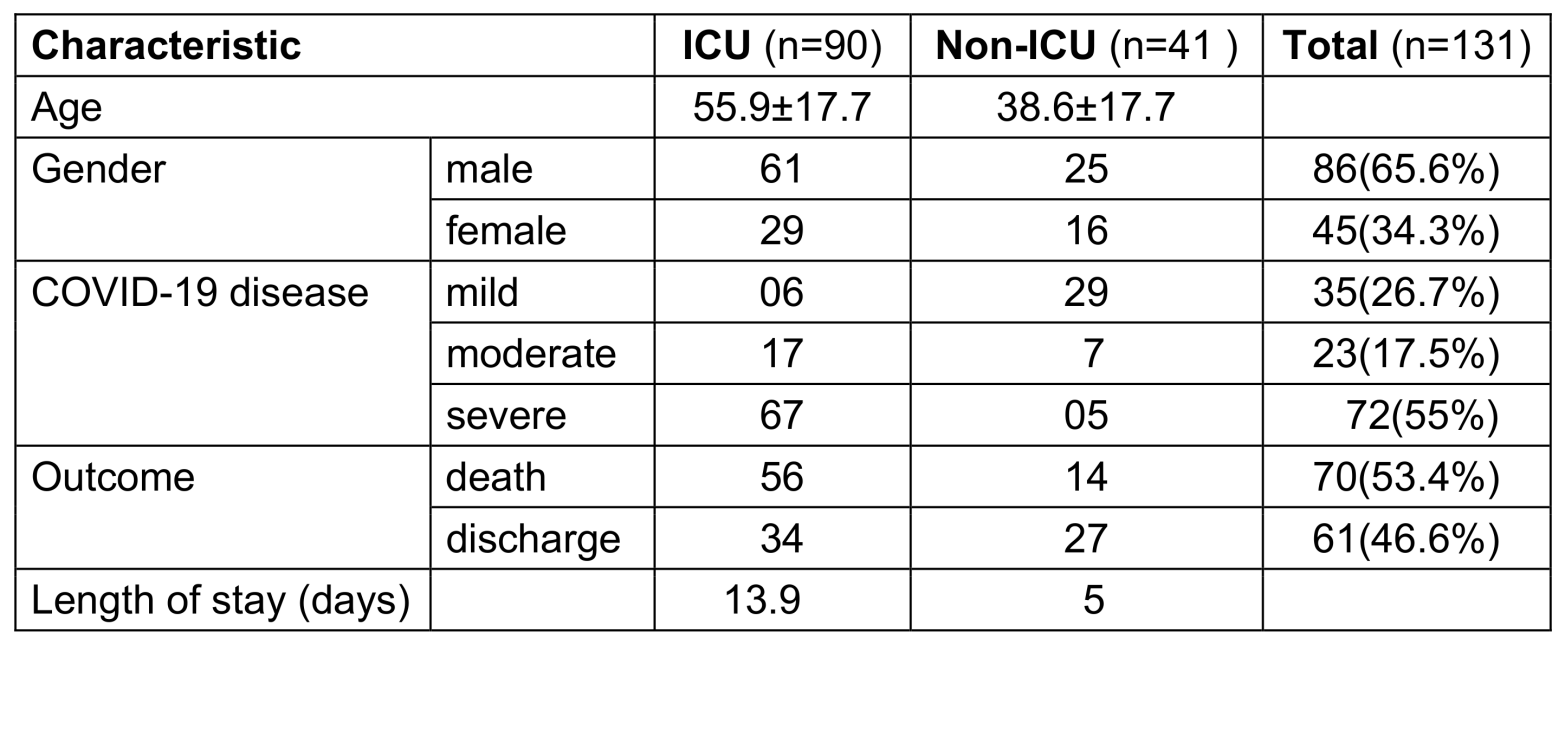
Demographic profile of 131 patients enrolled in the study

**Table 3:**
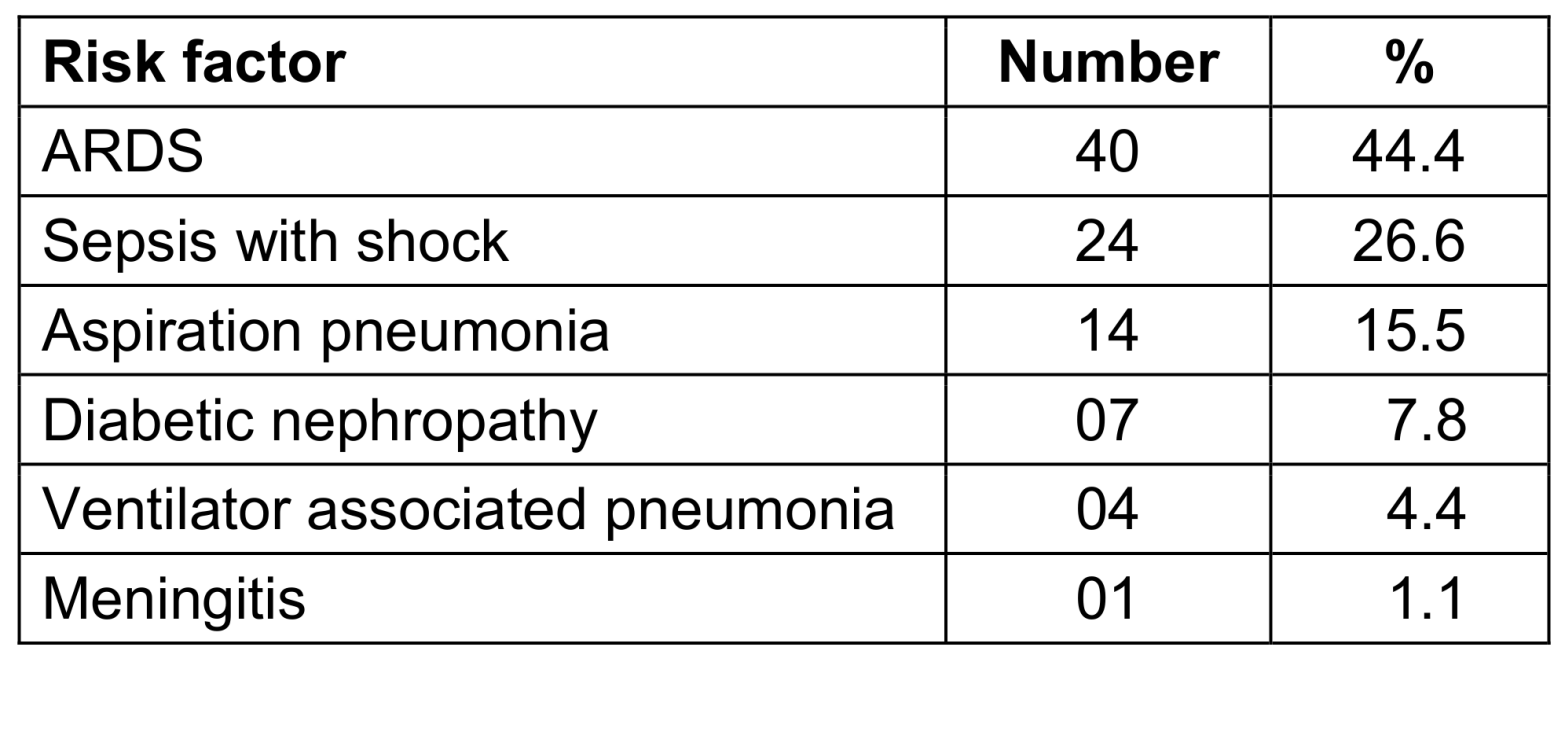
Clinical condition in COVID-19 patients with bacterial infections in ICU (n=90)

**Table 4:**
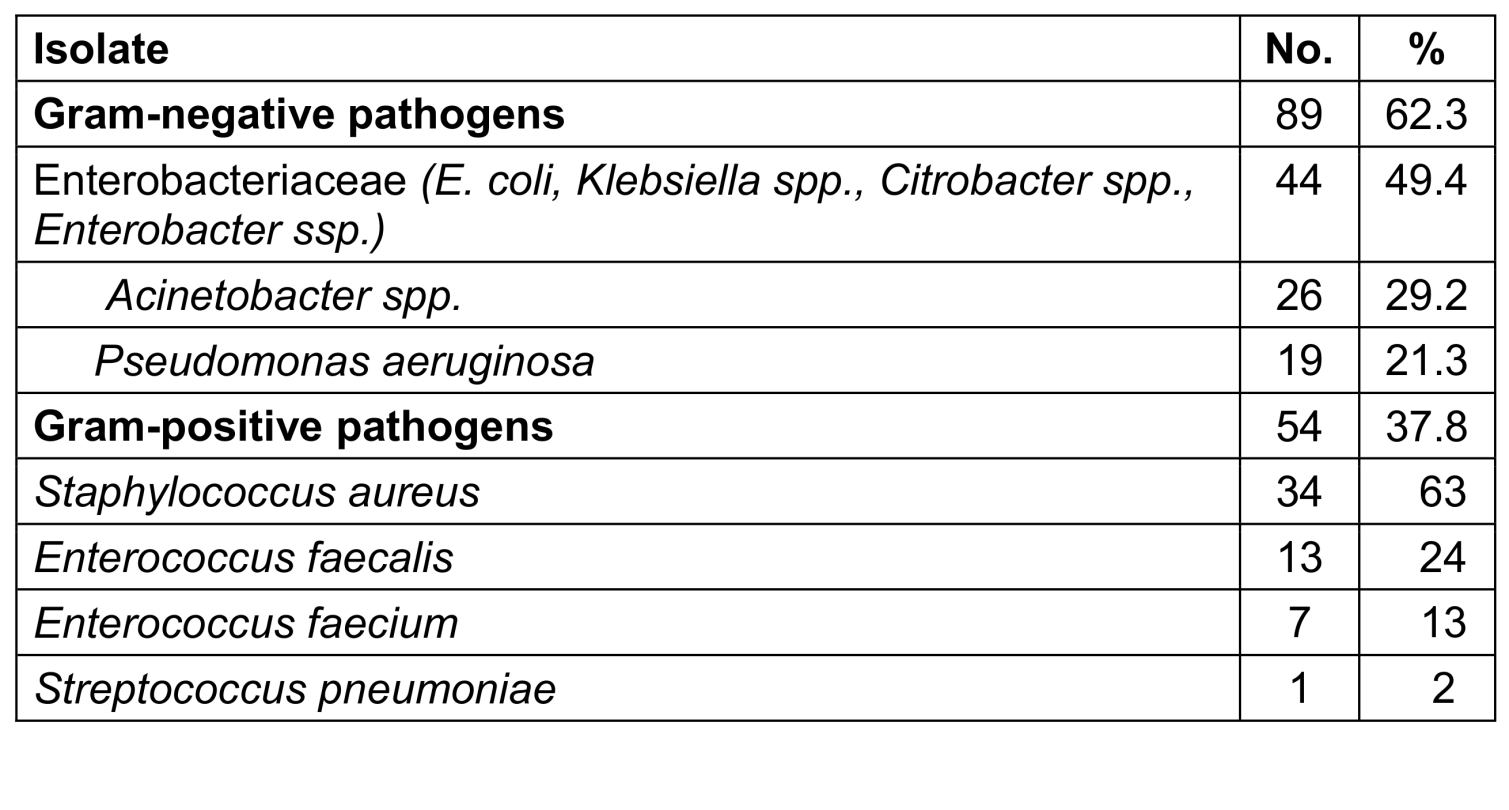
Etiology of bacterial infections in patients hospitalized with COVID-19 (n=143)

**Table 5:**
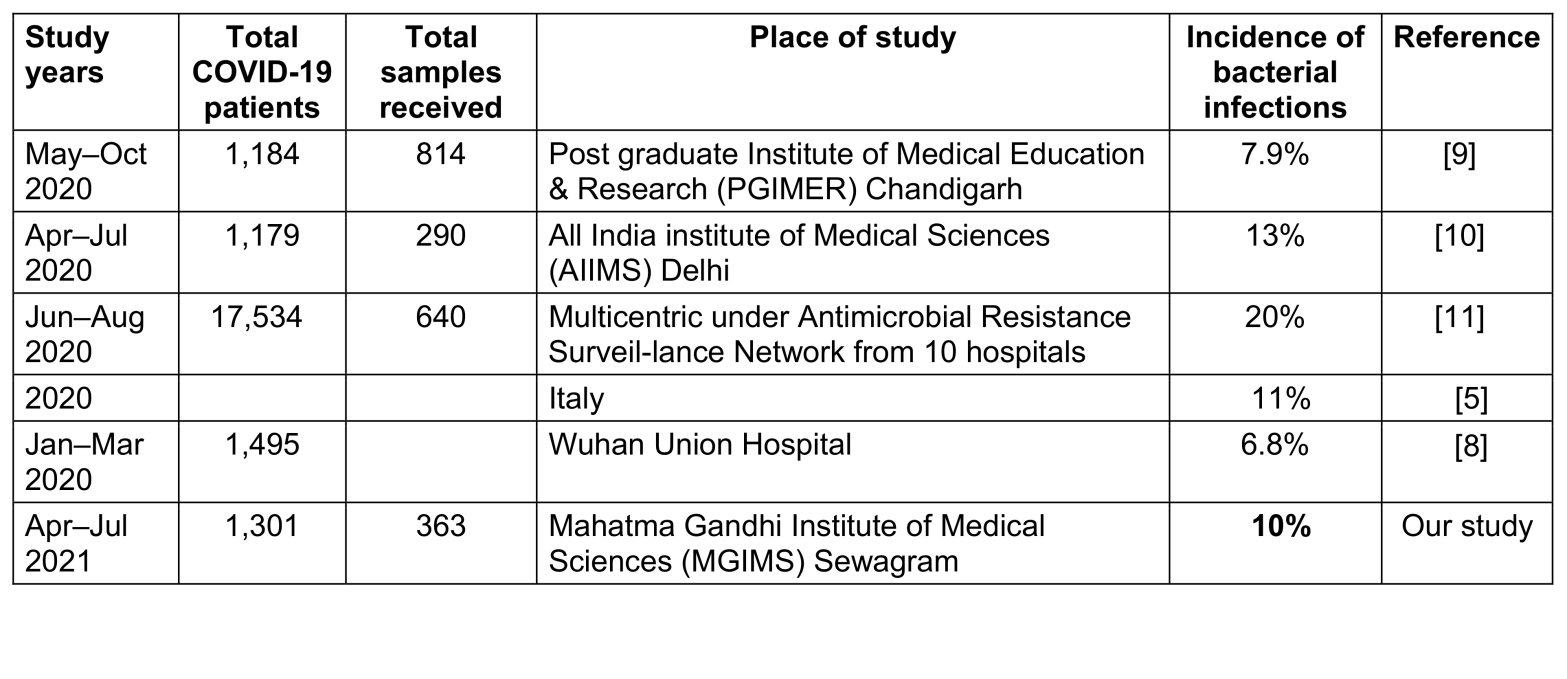
Rates of bacterial co-infections reported in the current medical literature for COVID-19 patients

## References

[1] Castagnoli R, Votto M, Licari A, Brambilla I, Bruno R, Perlini S, Rovida F, Baldanti F, Marseglia GL (2020). Severe Acute Respiratory Syndrome Coronavirus 2 (SARS-CoV-2) Infection in Children and Adolescents: A Systematic Review. JAMA Pediatr.

[2] Chen X, Liao B, Cheng L, Peng X, Xu X, Li Y, Hu T, Li J, Zhou X, Ren B (2020). The microbial coinfection in COVID-19. Appl Microbiol Biotechnol.

[3] Rice TW, Rubinson L, Uyeki TM, Vaughn FL, John BB, Miller RR, Higgs E, Randolph AG, Smoot BE, Thompson BT, NHLBI ARDS Network (2012). Critical illness from 2009 pandemic influenza A virus and bacterial coinfection in the United States. Crit Care Med.

[4] Shah NS, Greenberg JA, McNulty MC, Gregg KS, Riddell J, Mangino JE, Weber DM, Hebert CL, Marzec NS, Barron MA, Chaparro-Rojas F, Restrepo A, Hemmige V, Prasidthrathsint K, Cobb S, Herwaldt L, Raabe V, Cannavino CR, Hines AG, Bares SH, Antiporta PB, Scardina T, Patel U, Reid G, Mohazabnia P, Kachhdiya S, Le BM, Park CJ, Ostrowsky B, Robicsek A, Smith BA, Schied J, Bhatti MM, Mayer S, Sikka M, Murphy-Aguilu I, Patwari P, Abeles SR, Torriani FJ, Abbas Z, Toya S, Doktor K, Chakrabarti A, Doblecki-Lewis S, Looney DJ, David MZ (2016). Bacterial and viral co-infections complicating severe influenza: Incidence and impact among 507 U.S. patients, 2013-14. J Clin Virol.

[5] Huttner BD, Catho G, Pano-Pardo JR, Pulcini C, Schouten J (2020). COVID-19: don't neglect antimicrobial stewardship principles! Clin Microbiol Infect.

[6] Cox MJ, Loman N, Bogaert D, O'Grady J (2020). Co-infections: potentially lethal and unexplored in COVID-19. Lancet Microbe.

[7] Alhazzani W, Møller MH, Arabi YM, Loeb M, Gong MN, Fan E, Oczkowski S, Levy MM, Derde L, Dzierba A, Du B, Aboodi M, Wunsch H, Cecconi M, Koh Y, Chertow DS, Maitland K, Alshamsi F, Belley-Cote E, Greco M, Laundy M, Morgan JS, Kesecioglu J, McGeer A, Mermel L, Mammen MJ, Alexander PE, Arrington A, Centofanti JE, Citerio G, Baw B, Memish ZA, Hammond N, Hayden FG, Evans L, Rhodes A (2020). Surviving Sepsis Campaign: guidelines on the management of critically ill adults with Coronavirus Disease 2019 (COVID-19). Intensive Care Med.

[8] Li J, Wang J, Yang Y, Cai P, Cao J, Cai X, Zhang Y (2020). Etiology and antimicrobial resistance of secondary bacterial infections in patients hospitalized with COVID-19 in Wuhan, China: a retrospective analysis. Antimicrob Resist Infect Control.

[9] Vijay S, Bansal N, Rao BK, Veeraraghavan B, Rodrigues C, Wattal C, Goyal JP, Tadepalli K, Mathur P, Venkateswaran R, Venkatasubramanian R, Khadanga S, Bhattacharya S, Mukherjee S, Baveja S, Sistla S, Panda S, Walia K (2021). Secondary Infections in Hospitalized COVID-19 Patients: Indian Experience. Infect Drug Resist.

[10] Sharma B, Sreenivasan P, Biswal M, Mahajan V, Suri V, Singh Sehgal I, Ray P, Dutt Puri G, Bhalla A, Narayana Yaddanapudi L, Koushal V, Angrup A (2021). Bacterial coinfections and secondary infections in COVID-19 patients from a tertiary care hospital of northern India: Time to adhere to culture-based practices. Qatar Med J.

[11] Khurana S, Singh P, Sharad N, Kiro VV, Rastogi N, Lathwal A, Malhotra R, Trikha A, Mathur P (2021). Profile of co-infections & secondary infections in COVID-19 patients at a dedicated COVID-19 facility of a tertiary care Indian hospital: Implication on antimicrobial resistance. Indian J Med Microbiol.

